# High-Throughput Flow Injection Analysis–Mass Spectrometry (FIA-MS) Fingerprinting for the Authentication of Tea Application to the Detection of Teas Adulterated with Chicory

**DOI:** 10.3390/foods11142153

**Published:** 2022-07-20

**Authors:** Mònica Vilà, Àlex Bedmar, Javier Saurina, Oscar Núñez, Sònia Sentellas

**Affiliations:** 1 Department of Chemical Engineering and Analytical Chemistry, University of Barcelona, Martí i Franquès 1-11, E08028 Barcelona, Spain; monicavr2000@hotmail.com (M.V.); alexbedmar1999@gmail.com (À.B.); xavi.saurina@ub.edu (J.S.); sonia.sentellas@ub.edu (S.S.); 2 Research Institute in Food Nutrition and Food Safety, University of Barcelona, Recinte Torribera, Av. Prat de la Riba 171, Edifici de Recerca (Gaudí), Santa Coloma de Gramenet, E08921 Barcelona, Spain; 3 Serra Húnter Fellow, Generalitat de Catalunya, Rambla de Catalunya 19-21, E08007 Barcelona, Spain

**Keywords:** high-throughput analysis, FIA-MS, tea, chicory, fingerprinting, chemometrics, fraud detection, authentication

## Abstract

Tea is a broadly consumed beverage worldwide that is susceptible to fraudulent practices, including its adulteration with other plants such as chicory extracts. In the present work, a non-targeted high-throughput flow injection analysis-mass spectrometry (FIA-MS) fingerprinting methodology was employed to characterize and classify different varieties of tea (black, green, red, oolong, and white) and chicory extracts by principal component analysis (PCA) and partial least squares–discriminant analysis (PLS-DA). Detection and quantitation of frauds in black and green tea extracts adulterated with chicory were also evaluated as proofs of concept using partial least squares (PLS) regression. Overall, PLS-DA showed that FIA-MS fingerprints in both negative and positive ionization modes were excellent sample chemical descriptors to discriminate tea samples from chicory independently of the tea product variety as well as to classify and discriminate among some of the analyzed tea groups. The classification rate was 100% in all the paired cases—i.e., each tea product variety versus chicory—by PLS-DA calibration and prediction models showing their capability to assess tea authentication. The results obtained for chicory adulteration detection and quantitation using PLS were satisfactory in the two adulteration cases evaluated (green and black teas adulterated with chicory), with calibration, cross-validation, and prediction errors below 5.8%, 8.5%, and 16.4%, respectively. Thus, the non-targeted FIA-MS fingerprinting methodology demonstrated to be a high-throughput, cost-effective, simple, and reliable approach to assess tea authentication issues.

## 1. Introduction

Tea is one of the most consumed and popular beverages worldwide, obtained by the infusion of the leaves of the plant *Camellia sinensis*, which belongs to the Theaceae family [1]. The characteristic flavor and aroma of tea, together with the health-beneficial properties based on its antioxidant, anti-inflammatory, antimicrobial, anti-hypertensive, anticarcinogenic, neuroprotective, cholesterol-lowering, and thermogenic properties, are the main factors for the incorporation of this beverage by society in daily life [2,3,4,5]. Most of these properties are due to the presence of a great variety of bioactive substances. For example, teas are rich in polyphenolic compounds, such as catechins, gallic acid, flavonols, flavones, and proanthocyanidins, which are responsible for their important antioxidant capacity [6]. They also contain important amounts of non-protein amino acids, especially L-theanine and γ-aminobutyric acid, and some polyamines, such as methylxanthines. The main methylxanthine in tea is caffeine although theobromine and theophylline are also found in smaller quantities. Besides, teas that have undergone a total or partial fermentation process also contain specific derivatives of flavan-3-ols, i.e., theaflavins and thearubigins, formed during the oxidation process [7,8].

Tea can be classified into different varieties according to the fermentation process. In this way, black tea, a fully fermented and oxidized tea, is the most traditional one, representing 78% of the world production [9]. It is followed by green tea (22% of the world production), which is produced, without any fermentation, from dried tea leaves. The third fermented type is oolong tea, quite like black tea, but its fermentation is monitored to limit its oxidation to between 10 and 70%. Pu-erh tea, also known as red tea, is obtained from a specific tea plant variant (*Camellia sinensis* var. *assamica*) that grows only in Yunnan (China). It is obtained by chemical compositional changes of the dried tea leaves produced by bacteria under humid conditions. Finally, there is white tea, which is a very appreciated product variety produced from buds and younger tea leaves. In some regions, it is even shaded from the sunlight during its growing period to minimize the synthesis of chlorophyll. Then, immediately after harvest, the white tea leaves are carefully dried to prevent fermentation [10,11].

Nowadays, one of the main concerns worldwide is the growth of food fraud, which can be considered as an intentional substitution, addition, or alteration of food products through false claims about the product in order to reduce its price, increase its volume, and, above all, obtain illegal economic benefits [12,13]. Further, these fraudulent practices can suppose a serious risk to public health due to the possible presence of undeclared toxic substances or allergenic compounds. According to the European Commission, the most common type of fraud is adulteration, which can be accomplished by substituting nutrients with other components that are less valued or do not conform to standards or official labeling or by adding unknown and undeclared compounds [14]. The increased vulnerability to fraud is determined by the ease of adulterating certain types of products as well as the general availability of knowledge and techniques to carry out these fraudulent practices. As a result, it is sometimes difficult to recognize at a glance if a product has been adulterated [15]. Therefore, the development of analytical techniques to fight against fraudulent practices is required.

Tea is among the most adulterated beverages, together with coffee, fruit juices, and alcoholic drinks [12]. The most common adulterants used in tea are leather flakes, sand, dyes, coal tar, exhausted tea leaves, or leaves of lower-quality species. Other non-permitted materials used as tea adulterants are legume husks, borax, sodium carbonate, cereal starch, and chicory [16,17,18]. Focusing on chicory (*Cichorium intybus*), it is a perennial herbaceous plant that belongs to the Asteraceae family. It is cultivated worldwide, and its main use is in animal feed and the food industry, for example, as a supplement (if declared) in coffee and tea beverages or as a source for the production of inulin, a starch-like polysaccharide [19]. Several authors have reported the presence of chicory as adulterant in coffee and tea extracts [17]. For example, Deb Pal and Das [18] reported the presence of chicory as adulterant in black tea. They described that although chicory root gives a pleasant aroma when added to tea, it acts like a sedative on the central nervous system and could impair reaction time in some individuals. Besides, chicory can also trigger oral, cutaneous, and/or respiratory symptoms [20], and for that reason, its use as a non-declared adulterant in tea is prohibited.

Several targeted and non-targeted analytical methodologies have been reported to address tea authentication issues. Inductively coupled plasma-mass spectrometry (ICP-MS), isotope ratio mass spectrometry (IRMS), gas chromatography (GC), and liquid chromatography (LC) are among the techniques employed using targeted strategies for tea geographical characterization and authentication as well as to detect tea fraudulent practices [1,21,22,23,24]. In contrast, spectroscopic techniques, such as near-infrared (NIR), Fourier transform infrared (FTIR), and ultraviolet–visible (UV–vis), among others, have been widely employed as non-targeted fingerprinting strategies in tea authentication [25,26,27,28]. Non-targeted LC fingerprinting methodologies with UV–vis detection or coupled to low-resolution mass spectrometry (LC-LRMS) and high-resolution mass spectrometry (LC-HRMS) are also gaining popularity for the characterization, classification, and authentication of tea extracts [26,29,30,31,32]. However, many of these techniques tend to be time-consuming and require highly specialized personnel.

Nowadays, fast, reliable, cost-effective, and high-throughput methodologies able to cope with the high number of samples that need to be analyzed in the food authentication field are demanded. Among them, flow injection analysis–mass spectrometry (FIA-MS) is a simple method that offers high-throughput without compromising the sensitivity, precision, and accuracy [33]. With minimum sample manipulation and no separation at all, it is therefore gaining popularity to address food authentication and traceability issues [34,35,36,37]. For example, FIA-MS fingerprinting has been described for the authentication and quality assessment of nutraceuticals [34] and for the differentiation of three black cohosh species [35], in this last case by using HRMS. Recently, Campmajó et al. [36] demonstrated the feasibility of FIA-HRMS as a valuable tool to address food classification and authentication issues. Examples of its application to address the geographical origin classification of red wines and paprika, the distinction of olive oil from other vegetable oils, and the assessment of olive oil quality were described. Excellent classification accuracies were reached, and the use of HRMS allowed the characterization of the analyzed sample matrices by the putative identification of the most common ions.

We developed a non-targeted HPLC-UV-FLD fingerprinting methodology in combination with chemometrics to address classification and authentication of teas [33]. Although very acceptable results were obtained, the major handicap of the proposed methodology was the total analysis time, as a chromatographic separation of 25 min was required. After a preliminary study, we found that FIA-MS could be an appropriate technique to assess tea authentication issues [37]. Thus, in the present paper, a high-throughput methodology was developed to evaluate the applicability of non-targeted FIA-MS fingerprinting methodologies to the characterization, classification, and authentication of tea extracts (black, green, red, oolong, and white teas) as well as their feasibility to detect frauds and quantify adulterant percentages. FIA-MS fingerprints in both negative- and positive-ionization modes were used to address tea and chicory characterization and classification by exploratory principal component analysis (PCA) and supervised partial least squares–discriminant analysis (PLS-DA). Partial least square (PLS) regression was used to quantify the chicory percentages in adulterated black and green tea extracts.

## 2. Materials and Methods

### 2.1. Reagents and Chemicals

Methanol (Chromosolv^TM^ for HPLC, ≥99.9%) was obtained from PanReac AppliChem (Barcelona, Spain) and formic acid (≥98%) from Sigma-Aldrich (St. Louis, MO, USA). Water was purified with an Elix 3 coupled to a Milli-Q system from Millipore Corporation (Millipore, Bedford, MA, USA).

A commercial natural mineral water with weak mineralization purchased from Eroski (Barcelona, Spain), as proposed in a previously published work [32], was used for the preparation of tea and chicory extracts. The water chemical composition was as follows: 402 mg/L of dry residue at 180 °C, 326 mg/L of hydrogen carbonate, 44 mg/L of chloride, 85 mg/L of calcium, 28 mg/L of magnesium, 18 mg/L of sodium, and 8 mg/L of silica.

### 2.2. Samples and Sample Treatment

A total of 101 teas of different varieties and 20 commercial chicory samples (Table 1) purchased from several supermarkets in Barcelona (Spain) were analyzed for characterization and classification purposes. A more detailed description of samples (commercial name and country of origin) is provided in Table S1 (Supplementary Materials).

Samples were prepared by extraction following a previously described procedure [32]. Briefly, 0.5 g of tea or chicory samples were extracted with 25 mL of hot water in polypropylene tubes (Serviquimia, Barcelona, Spain). The mixture was shaken for one minute in a Vortex (Stuart, Stone, UK) to assure the quantitative extraction, and centrifuged for 5 min at 3500 rpm using a Rotanta 460 RS centrifuge (Hettich, Tuttlingen, Germany). The extracts were then filtered with 0.45 µm nylon filters (discarding the first mL) into amber glass injection vials and kept at 4 °C until their analysis by FIA-MS.

A quality control (QC) solution was also prepared by mixing 50 µL of each one of the obtained aqueous tea and chicory extracts. The purpose of this QC (composed sample) was to evaluate the reproducibility of the proposed methodologies as well as the robustness of the chemometric results obtained.

The detection and quantitation of chicory adulterations in tea extracts were evaluated by studying two cases based on black and green teas adulterated with chicory. For each adulteration case, different blends, following the same strategy previously proposed [32], were prepared to build the corresponding PLS regression calibration and validation sets (Table 2).

Each adulteration level was prepared in quintuplicate, obtaining 55 sample extracts for each one of the two adulteration cases under study. With the aim to cover the differences among teas of the same product variety (black or green tea) as well as among chicories, ten different tea samples within each product variety and four different chicory samples were used for the preparation of the mixtures indicated in Table 2. However, for the preparation of the blended mixtures, the ten tea-product variety samples were not pooled, and neither were the four chicory samples, but a given tea-product variety sample was blended with a given chicory sample. Therefore, the five replicates of each blended level used were obtained with different tea/chicory sample combinations. The information showing how the samples were blended is summarized in Table S2 (Supplementary Materials). An additional blended tea and chicory sample at 50% level was used as the QC sample.

### 2.3. Instrumentation

Flow injection analysis–mass spectrometry (FIA-MS) was performed using an Agilent 1100 Series HPLC instrument (Waldbronn, Germany) coupled to an AB Sciex 4000 QTrap hybrid triple quadrupole/linear ion trap mass spectrometer (AB Sciex, Framingham, MA, USA). FIA-MS experiments were carried out by injecting 10 µL of sample on a 50:50 (*v*/*v*) mixture of methanol and water acidified with 0.1% formic acid (*v*/*v*) as the carrier pumped at a flow rate of 150 µL min^−1^. FIA-MS fingerprints, in negative and positive electrospray ionization (ESI), were obtained in EMS (Enhance Mass Spectrometry) mode (*m*/*z* 100–550). The ion spray voltage was set at 2500 V and −2500 V in positive and negative polarities, respectively, and the source temperature was kept at 400 °C. Furthermore, a declustering potential (DP) of 80 V (positive or negative depending on the ionization mode) was employed. Nitrogen was used as nebulizer and auxiliary gas and was set at 10, 50, and 50 arbitrary units for the curtain gas, the ion source gas 1, and the ion source gas 2, respectively. Total FIA-MS analysis time was 1.5 min.

### 2.4. Data Analysis

#### 2.4.1. Data Matrices

For both classification/characterization and adulteration studies, raw data obtained by FIA-MS were initially processed with the MSConvert free software to transition them into an mzML output format (ProteoWizard, Palo Alto, CA, USA). We applied 32 bits as binary encoding precision and Threshold Peak Filter for data simplification. The absolute intensity was defined as threshold at a value of 10,000 counts.

The mzML files were transformed into the working data matrices containing the FIA-MS fingerprints using the mzMine 3 software, in which ion signal intensities were arranged as a function of samples in rows and *m*/*z* variables in columns [38]. The first step was the wavelet transform mass detection, which generates mass lists for each of the scans acquired on a sample. Thus, the wavelet transform was selected as the mass detector since it is the most useful for low-resolution mass spectrometry. A peak time range of 0.00–1.48 min was set as well as the corresponding polarity. A noise level of 4.0 × 10^4^, a scale level of 3, and a wavelet window size of 30% were also considered. The next step was to remove false signals with the FTMS shoulder peak filter by setting a Gaussian peak model function and a mass resolution of 70,000. Then, to extract ion chromatograms for masses that were detected continuously for a given time, the ADAP chromatogram builder was used, defining a minimum scan group size of 5, a group intensity threshold of 4.0 × 10^4^, a highest minimum intensity of 1.0 × 10^4^, and an *m*/*z* tolerance of 5000 ppm, which is equivalent to 1 *m*/*z*. Lastly, the Join Aligner was performed to allow matching of the masses detected across the analyzed samples; hence, an *m*/*z* tolerance of 5000 ppm, a weight for *m*/*z* of 80, a retention time tolerance of 2 min, and a weight for RT and a mobility weight of 1 were defined, thus obtaining the aligned feature list that was finally exported as CSV format and ready for subsequent chemometric analysis. The dimensions of the obtained data matrices (samples + QCs × variables) were (138 × 320) and (138 × 339) for negative- and positive-ionization mode, respectively.

#### 2.4.2. Chemometric Data Analysis

The obtained data matrices were then submitted to PCA, PLS-DA, and PLS using SOLO 8.6 chemometric software from Eigenvector Research (Manson, WA, USA). Details of the theoretical background of these chemometric methods are reported in reference [39]. The X-data matrix consisted of the FIA-MS fingerprints (peak signal as a function of *m*/*z* values and run time) obtained in negative- or positive-ionization modes. Y-data matrices were also built coding the sample class in PLS-DA or the chicory percentage in PLS.

In PCA and PLS-DA, FIA-MS fingerprints were autoscaled to eliminate differences in their magnitude and amplitude scales. In this way, all the variables became equally weighted. VIP (Variable Importance in Projection) treatment was applied to PLS-DA to reduce the number of *m*/*z* variables by selecting only the most descriptive ones: here, those that scored above 1 in the VIP analysis. The number of latent variables (LVs) in PLS-DA and PLS was chosen by cross-validation (CV) using a Venetian Blind approach with 10 splits, thus leading to the corresponding 10 sub-validation experiments to calculate the overall error. Furthermore, in PLS-DA, 60% of the samples (randomly selected) were taken for calibration and the remaining 40% for prediction.

## 3. Results and Discussion

As commented in the introduction section, we previously developed a HPLC-UV-FLD fingerprinting strategy that provided very acceptable results for the classification and authentication of tea extracts but required a high total analysis time (chromatographic separation of 25 min) [32]. In this work, we aimed to develop a high-throughput FIA-MS fingerprinting methodology for characterization, classification and authentication of tea extracts and the detection of chicory adulteration in tea as an alternative to the previously developed method.

### 3.1. High-Throughput FIA-MS Fingerprints

**Figure 1:**
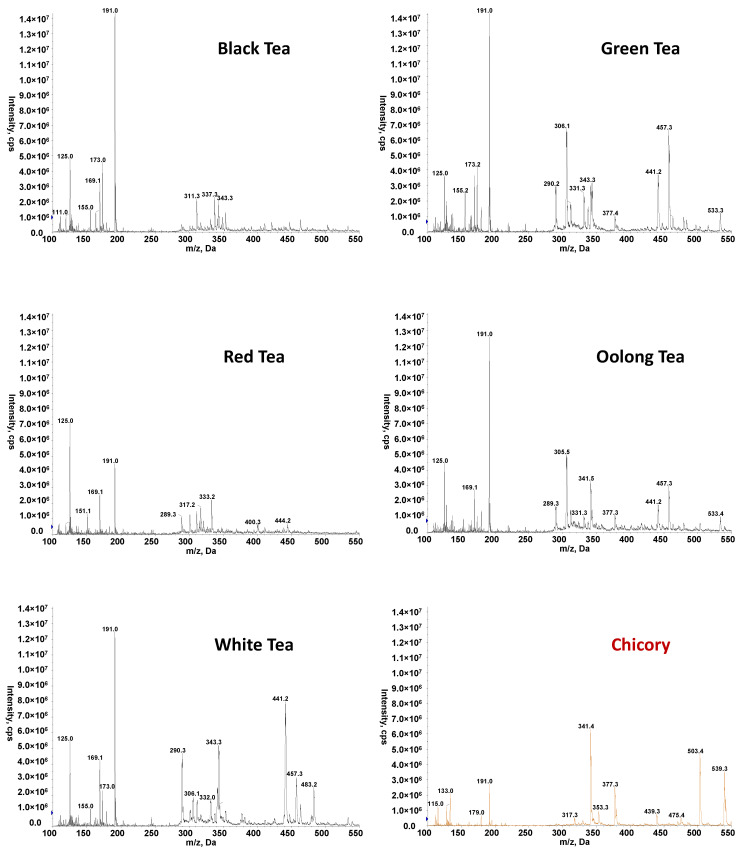
Non-targeted FIA-ESI(−)-MS fingerprints for selected tea and chicory samples.

**Figure 2:**
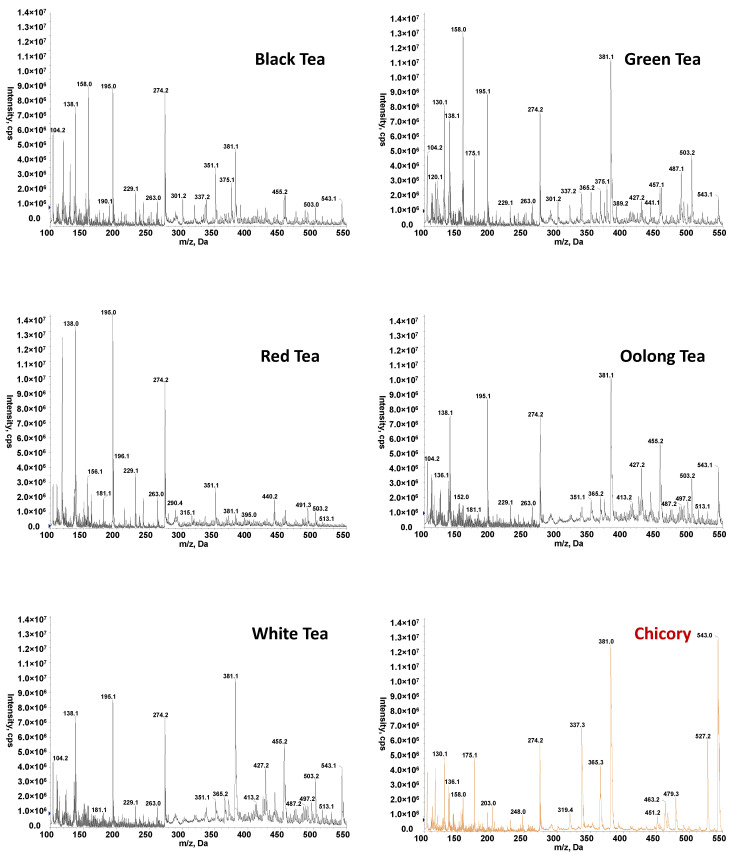
Non-targeted FIA-ESI(+)-MS fingerprints for selected tea and chicory samples.

After brewing the tea or chicory with hot water, samples were analyzed by FIA-MS, injecting 10 µL of each extract into a carrier of methanol:0.1% formic acid aqueous solution 50:50 (*v*/*v*) at 150 µL min^−1^. FIA-MS fingerprints were registered in both negative and positive-ESI mode and using a hybrid triple quadrupole/linear ion trap mass spectrometer (QTrap) working in EMS mode (*m*/*z* 100–550). The total analysis time per sample was established in 1.5 min. Figure 1 and Figure 2 show the obtained FIA-MS fingerprints in negative- and positive-ionization mode, respectively, for the selected teas (black, green, red, oolong, and white varieties) and chicory samples. As can be seen in the figures, independently of the tea product variety and chicory sample, FIA-ESI(+)-MS fingerprints tend to be richer in comparison to those in negative-ionization mode. Important dissimilarities regarding the detected ions and their intensities were also observed between the different varieties of tea and chicory samples (both in positive and negative modes). Irrespectively of the nature of the chemicals responsible for the signals detected on the obtained fingerprints, the significant differences observed between tea and chicory samples and, to some extent, also between the different tea varieties and the fact that these observed characteristics are also reproducible within each sample group suggested that the proposed FIA-MS fingerprints could be appropriate for tea authentication using multivariate chemometric methods.

### 3.2. Exploratory Principal Component Analysis

**Figure 3:**
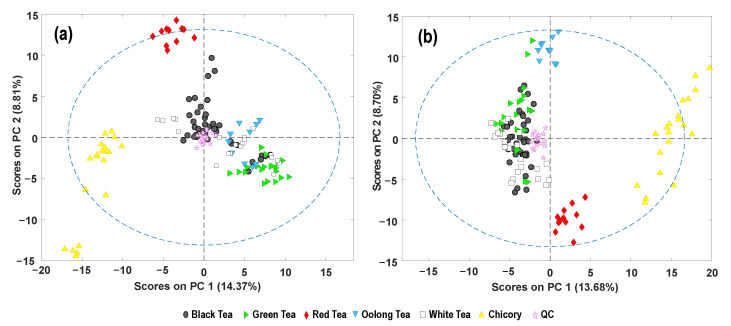
Exploratory PCA score plots of PC1 vs. PC2 when using FIA-MS fingerprints registered in (**a**) negative- and (**b**) positive-ionization mode as sample chemical descriptors.

As a first attempt to address tea authentication, a PCA model was built as an exploratory method to study tea and chicory sample distribution based on the fingerprinting features. Moreover, data reproducibility as well as the robustness of the chemometric results were assessed from the observed QCs’ behavior. The score plots of PC1 versus PC2 using FIA-MS fingerprints using both ionization modes are shown in Figure 3. QCs appeared clustered in the center of the score plots, demonstrating both the reproducibility of the proposed FIA-MS methodology and the robustness of the obtained chemical results.

Tea and chicory were grouped according to their sample class (tea or chicory) or the tea-product variety. Independently of the FIA-MS ionization mode, chicory samples are perfectly separated and discriminated from tea samples based on PC1, being grouped at the left area of the plot (with negative-PC1 values) and the right area of the plot (with positive PC1 values) for FIA-ESI(−)-MS and FIA-ESI(+)-MS fingerprints, respectively. Regarding the different tea varieties under study, FIA-ESI(+)-MS fingerprints (Figure 3b) demonstrated higher separation and discrimination capabilities than the one attained by using negative-ionization mode. In this case, two tea varieties (red and oolong teas) are perfectly separated from the others (clearly discriminated by PC2), while only red teas are set apart in the case of FIA-ESI(−)-MS fingerprints. All the other tea varieties tend to be overlapped although higher sample distribution in the PCA plot is observed with the negative-ionization mode.

### 3.3. Supervised Partial Least Squares–Discriminant Analysis

The characterization and classification of tea and chicory samples were also evaluated by PLS-DA, and the obtained results are summarized in Table 3, in which multiclass cross-validated classification models were assessed. The optimal number of LVs for establishing such models was 7 and 6 for positive and negative ionization, respectively.

As can be seen, the overall performance was slightly superior, and chicory and red and oolong teas were perfectly discriminated from the other classes, with no confusion with the other sample types. For black, green, and white teas, some misclassifications were encountered although, in any case, classification rates were always higher than 94%. The PLS-DA cross-validated model also provided a perfect distinction of chicory and red tea samples from the other groups. In contrast, some confusion was detected among black, green, oolong, and white tea. Regardless, classification rates are 90% or better.

Even though FIA is did not perform any chromatographic separation of compounds, better classification results were obtained with the proposed FIA-MS fingerprinting methodology in comparison to the one previously reported using HPLC-UV and HPLC-FLD fingerprints [32]. In that previous work, some overlapping between chicory samples and some tea varieties was observed when using PLS-DA. Further, better tea-product variety classification was also accomplished by FIA-MS. This is a noteworthy result, as FIA-MS fingerprinting is much faster, with a total instrumental analysis time of 1.5 min per sample in comparison to the 25 min per sample required by HPLC-UV or HPLC-FLD fingerprinting, thus making FIA-MS an ideal high-throughput screening methodology for authentication purposes.

**Figure 4:**
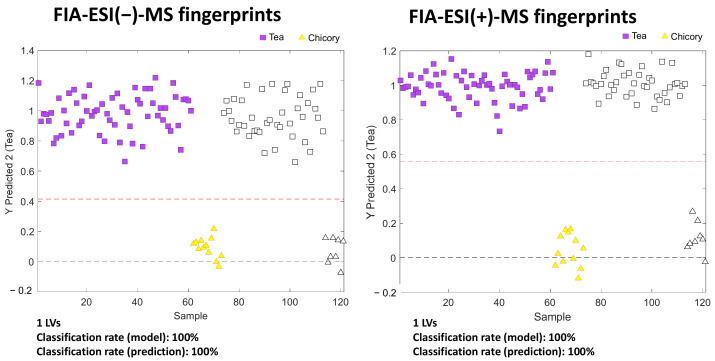
Validation of the paired PLS-DA models of all tea product varieties versus chicory when using FIA-MS fingerprints from negative- and positive-ionization modes as the sample chemical descriptors. Red line means the separation threshold between classes. Filled and empty symbols correspond to the calibration and validation sets, respectively.

Paired PLS-DA models were constructed for the classification of all the tea-product varieties in front of chicory using high-throughput FIA-MS fingerprints. A total of 60% of the samples (selected randomly) were used for calibration and the remaining 40% as “unknown samples” for validation and prediction. The obtained validation results are depicted in Figure 4.

Classification rates for both calibration and prediction were excellent (100% rate). For comparison with a previous published method [32], the validation of paired PLS-DA models of each tea product versus chicory were provided in Figure S1 (Supplementary Materials). In general, only one LVs was needed (except for white tea vs. chicory, where two LV were used). Again, these results were much better than the ones previously reported when using HPLC-UV and HPLC-FLD fingerprints for tea versus chicory classification, where some tea varieties showed lower classification rates, such as green and oolong tea with HPLC-FLD data (96.4% and 95.5%, respectively, for calibration) or white and oolong tea with HPLC-UV data (77.8% and 88.9%, respectively, for prediction) [32]. Consequently, both FIA-MS fingerprints in negative- and positive-ionization modes can be proposed to address tea extract classification and authentication when adulterated with chicory samples.

### 3.4. Detection and Quantitation of Chicory Adulterations in Tea by Partial Least Squares Regression

The capacity of the FIA-MS fingerprinting methodologies to detect tea frauds and quantify tea adulteration levels was evaluated by PLS regression from two representative cases: black tea and green tea, both adulterated with different percentages of chicory. These examples should be understood as a proof-of-concept study in which the possibilities of detecting and quantifying tea adulterations with chicory were assessed. We decided to focus on the black and green tea systems since these are the most consumed tea types worldwide; moreover, the number of samples available from these groups was wider. Conclusions extracted from these model cases reasonably supported the validity of our FIA-MS approach for dealing with fraud cases of oolong, red, and white teas, in which the number of samples available in our study was more limited.

For each one of the adulteration cases, two independent sets of samples with different chicory percentages were prepared for calibration and validation purposes, as summarized in Table 2. Moreover, to cover a wide range of tea samples within the same tea product variety (black or green) as well as with the chicory samples, for the preparation of the blended mixtures of ten different tea samples (different production regions) of each product variety (black or green) and four different chicory samples were employed (Table S2, Supplementary Materials). The blended samples (prepared in quintuplicate) were then extracted with hot water, and extracts were analyzed randomly by FIA-MS in both negative- and positive-ionization modes. The obtained FIA-MS fingerprints were used as the data.

**Figure 5:**
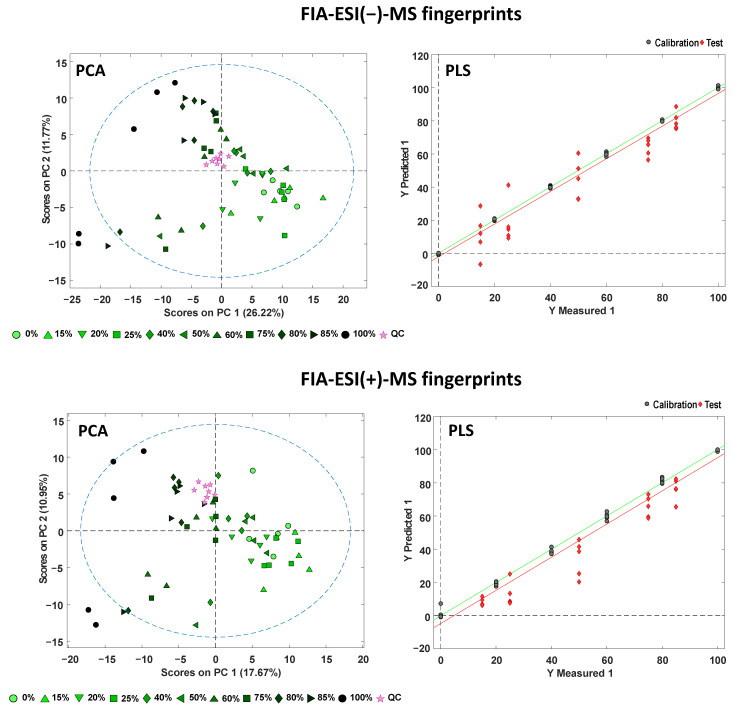
PCA score plots of PC1 versus PC2 and PLS scatter plots of measured versus predicted percentages of chicory for adulterated green tea by employing FIA-MS fingerprints in negative- and positive-ionization mode.

A preliminary study by PCA of all the blended samples for each adulteration case was performed to evaluate the behavior of the QCs and the sample distribution. Figure 5 shows, as an example, the PCA score plots of PC1 versus PC2 for the green tea adulterated with chicory case when using FIA-MS fingerprints in both negative- and positive-ionization mode as sample chemical descriptors. The same data for the black tea adulterated with chicory case is provided in Figure S2 of the Supplementary Materials.

In both cases, QCs appeared perfectly grouped, demonstrating again the good reproducibility of the proposed methodology and confirming the robustness of the obtained chemometric results. Besides, blended samples tend to be distributed within the score plots according to the chicory percentage through PC1, with pure tea samples and those with low adulteration levels located at the right area (with positive PC1 values) and pure chicory samples and tea adulterated at high concentration levels located in the opposite area (left part of the plot, exhibiting negative PC1 values). The dispersion of some blended groups is because the blended samples were prepared with both tea and chicory samples from different production regions (details in Tables S1 and S2 of Supplementary Materials), to cover the highest possible sample variability for possible fraudulent practices, as this is a more realistic situation than only employing one specific tea and one specific chicory sample for the adulteration study.

PLS regression models were built with the calibration blended samples set and the validation blended samples set quantified as unknown samples. The scatter PLS plots of Y measured versus Y predicted obtained with both FIA-MS fingerprints (in negative- and positive-ionization modes) for the green tea adulterated with chicory are shown in Figure 5 (same data for the black tea adulterated with chicory case is provided in Figure S2 of the Supplementary Materials). The number of LVs required to build each model, the determination coefficients (R^2^), as well as the calibration errors (RMSEC, root mean squares error calibration), the cross-validation error (RMSECV, root mean squarer error cross-validation), and the prediction error (RMSEP, root mean squares error prediction) obtained for each PLS model are summarized in Table 4. These PLS models were able to predict chicory concentrations above 2% in both black and green tea systems with a reasonable accuracy. Moreover, a scenario of adulterations using low(er) percentages of chicory is of little interest from a practical point of view since the economic gains associated with adding such small quantities of chicory would be rather scarce.

As can be seen, quite satisfactory results were obtained for both adulteration cases under study even considering the great variability of the samples. Acceptable calibration, cross-validation, and prediction regression coefficients were, in general, obtained. R^2^ values higher than 0.944 were obtained except for FIA-MS fingerprints in negative-ionization mode for the adulterated green tea and black tea. In addition, very acceptable calibration and cross-validation errors were obtained (always below 10%), while higher values for prediction errors were observed for the test set, with values ranging from ca. 8 to 16%. Comparing both FIA-MS fingerprinting methodologies, the obtained data are similar or slightly better with the positive-ionization mode, which can be attributed to their highest discrimination capacity between tea varieties and chicory observed also in the classification PLS-DA studies (Figure 4).

In any case, the FIA-MS fingerprints in both negative- and positive-ionization modes result in good sample descriptors to address tea and chicory classification and to quantify tea adulterations with chicory.

## 4. Conclusions

Non-targeted high-throughput FIA-MS fingerprints in both negative- and positive-ionization modes have shown to be excellent sample chemical descriptors to assess the characterization, classification, and authentication of tea samples of different varieties and chicory.

Both PCA and PLS-DA provided perfect discrimination of chicory samples against the five tea varieties under study. In addition, the classification and authentication capacity of the proposed high-throughput FIA-MS fingerprinting methodologies was demonstrated by the validation of paired tea versus chicory PLS-DA models. Independently of the FIA-MS fingerprints (negative- or positive-ionization mode), classification rates were 100% for both calibration and prediction.

PLS was applied to two adulteration cases based on black and green teas adulterated with chicory. Very acceptable PLS results for quantitation were also accomplished, with calibration, cross-validation, and prediction errors below 5.8%, 8.5%, and 16.4%, respectively. These results can be considered very good considering that both calibration and prediction set of samples were prepared by employing a high number of tea and chicory samples with different geographical origins, aiming to cover a higher variability range of possible adulteration cases.

In conclusion, the proposed non-targeted FIA-MS fingerprinting methods can be used as high-throughput, simple, cost-effective, and reliable screening methodologies to classify and authenticate tea and chicory samples and to prevent fraudulent practices using chicory as the adulterant.

## Figures and Tables

**Table 1:** Number and varieties of the analyzed tea and chicory samples.

Sample Class	Sample Type (Codification)	Total Number of Samples
Tea	Black tea (B)	39
	Green tea (G)	20
	Oolong tea (O)	10
	Red tea (R)	12
	White tea (W)	20
Chicory	Chicory (C)	20

**Table 2:** Black or green tea and chicory blends used in the study of adulterations by PLS (*n* = 5 for each mixture).

	Tea (%)	Chicory (%)		Tea (%)	Chicory (%)
**Calibration set**	100	0	**Validation set**	85	15
	80	20		75	25
	60	40		50	50
	40	60		25	75
	20	80		15	85
	0	100			

**Table 3:** PLS-DA cross-validated results for multiclass models from both positive- and negative-ionization data sets.

*FIA-MS: Positive-Ionization Mode*			
PLS-DA Model with 7 LVs			
**Class**	**Sensitivity (%) ^a^**	**Specificity (%) ^b^**	**False-positive remarks**
Black tea	94.8	100	None
Green tea	95.0	95.0	Black tea (1); white tea (4)
Oolong tea	100	100	None
Red tea	100	100	None
White tea	100	96.0	Black tea (2); green tea (2)
Chicory	100	100	None
***FIA-MS: Positive-Ionization Mode***			
**PLS-DA Model with 6 LVs**			
**Class**	**Sensitivity (%) ^a^**	**Specificity (%) ^b^**	**False-positive remarks**
Black tea	92.3	98.7	White tea (1)
Green tea	95.0	94.1	Black tea (1); oolong tea (2); white tea (3)
Oolong tea	90.0	96.4	Green tea (4)
Red tea	100	100	None
White tea	90.0	98.0	Green tea (2)
Chicory	100	100	None

^a^, true-positive rate in percentage; ^b^, true-negative rate in percentage.

**Table 4:** PLS regression results for the two adulteration cases under study.

*Green Tea Adulterated with Chicory*							
	LVs	Calibration (R^2^)	Cross-Validation (R^2^)	Prediction (R^2^)	RMSEC (%)	RMSECV (%)	RMSEP (%)
**FIA-ESI(−)-MS fingerprints**	4	1.000	0.965	0.881	0.7	6.7	11.5
**FIA-ESI(+)-MS fingerprints**	4	0.997	0.960	0.935	2.0	7.2	12.8
***Black Tea Adulterated with Chicory***							
	**LVs**	**Calibration (R^2^)**	**Cross-Validation (R^2^)**	**Prediction (R^2^)**	**RMSEC (%)**	**RMSECV (%)**	**RMSEP (%)**
**FIA-ESI(−)-MS fingerprints**	2	0.974	0.949	0.770	5.5	7.9	16.4
**FIA-ESI(+)-MS fingerprints**	2	0.971	0.944	0.946	5.8	8.5	7.8

## Data Availability

Data is contained within the article or Supplementary Materials.

## Supplementary Materials

The following supporting information can be downloaded at: https://www.mdpi.com/article/10.3390/foods11142153/s1, Figure S1: Validation of the paired PLS-DA models of each tea-product variety versus chicory when using FIA-MS fingerprints from negative- and positive-ionization modes as the sample chemical descriptors. Red line establishes the separation threshold between both classes. Filled symbols correspond to the calibration set and empty symbols correspond to the validation set (unknown samples for prediction purposes); Figure S2: PCA score plots of PC1 versus PC2 depicting the distribution of both calibration and validation sets and the PLS scatter plots of measured versus predicted percentages of adulterant (chicory) for the black tea adulterated with chicory case by employing FIA-MS fingerprints in negative- and positive-ionization mode as sample chemical descriptors; Table S1: Information about the analyzed samples; Table S2: Blends of black tea samples adulterated with chicory according to the percentage of chicory employed. B1 to B10 refer to the ten different black tea product variety samples, and C1 to C4 refer to the four different chicory samples employed. The same procedure was applied with green tea samples.

## References

[1] Inarejos-García A.M., Helbig I., Klette P., Weber S., Maeder J., Morlock G.E. (2021). Authentication of Commercial Powdered Tea Extracts (*Camellia sinensis* L.) by Gas Chromatography. ACS Food Sci. Technol..

[2] Sanlier N., Gokcen B.B., Altuğ M. (2018). Tea consumption and disease correlations. Trends Food Sci. Technol..

[3] Jie G., Lin Z., Zhang L., Lv H., He P., Zhao B. (2006). Free radical scavenging effect of Pu-erh tea extracts and their protective effect on oxidative damage in human fibroblast cells. J. Agric. Food Chem..

[4] Jang M.H., Piao X.L., Kim J.M., Kwon S.W., Park J.H. (2009). Antilipogenic Effect of Green Tea Extract in C57BL/6J-Lepob/ob Mice. Phyther. Res..

[5] Okello E.J., Savelev S.U., Perry E.K. (2004). In vitro anti-β-secretase and dual anti-cholinesterase activities of *Camellia sinensis* L. (tea) relevant to treatment of dementia. Phyther. Res..

[6] Spáčil Z., Nováková L., Solich P. (2010). Comparison of positive and negative ion detection of tea catechins using tandem mass spectrometry and ultra high performance liquid chromatography. Food Chem..

[7] Da Silva Pinto M. (2013). Tea: A new perspective on health benefits. Food Res. Int..

[8] Sang S., Caballero B., Finglas P.M., Toldrá F. (2016). Tea: Chemistry and Processing. Encyclopedia of Food and Health.

[9] Cabrera C., Artacho R., Giménez R. (2006). Beneficial Effects of Green Tea—A Review. J. Am. Coll. Nutr..

[10] Liang Y., Zhang L., Lu J. (2005). A study on chemical estimation of pu-erh tea quality. J. Sci. Food Agric..

[11] Chen Q., Zhao J., Fang C.H., Wang D. (2007). Feasibility study on identification of green, black and Oolong teas using near-infrared reflectance spectroscopy based on support vector machine (SVM). Spectrochim. Acta-Part A Mol. Biomol. Spectrosc..

[12] Kamiloglu S. (2019). Authenticity and traceability in beverages. Food Chem..

[13] Zambonin C. (2021). Maldi-tof mass spectrometry applications for food fraud detection. Appl. Sci..

[14] European Comission Food Fraud: What Does It Mean?. https://ec.europa.eu/food/safety/food-fraud/whatdoes-it-mean_en.

[15] Van Ruth S.M., Huisman W., Luning P.A. (2017). Food fraud vulnerability and its key factors. Trends Food Sci. Technol..

[16] Lagiotis G., Stavridou E., Bosmali I., Osathanunkul M., Haider N., Madesis P. (2020). Detection and quantification of cashew in commercial tea products using High Resolution Melting (HRM) analysis. J. Food Sci..

[17] Sharma K., Singh S., Tanwar K. (2020). Recognition and Evaluation of Authenticity of Tea and Coffee. Int. J. Adv. Res. Sci. Commun. Technol..

[18] Pal A.D., Das T. (2018). Analysis of adulteration in black tea. Int. J. Biol. Res..

[19] Perović J., Tumbas Šaponjac V., Kojić J., Krulj J., Moreno D.A., García-Viguera C., Bodroža-Solarov M., Ilić N. (2021). Chicory (*Cichorium intybus* L.) as a food Ingredient–Nutritional composition, bioactivity, safety, and health claims: A review. Food Chem..

[20] Cadot P., Kochuyt A.M., Van Ree R., Ceuppens J.L. (2003). Oral allergy syndrome to chicory associated with birch pollen allergy. Int. Arch. Allergy Immunol..

[21] Ma G., Zhang Y., Zhang J., Wang G., Chen L., Zhang M., Liu T., Liu X., Lu C. (2016). Determining the geographical origin of Chinese green tea by linear discriminant analysis of trace metals and rare earth elements: Taking Dongting Biluochun as an example. Food Control..

[22] Liu W., Chen Y., Liao R., Zhao J., Yang H., Wang F. (2021). Authentication of the geographical origin of Guizhou green tea using stable isotope and mineral element signatures combined with chemometric analysis. Food Control..

[23] Xia W., Li Z., Yu C.C., Liu Z., Nie J., Li C., Shao S., Zhang Y., Rogers K.M., Yuan Y. (2021). Understanding processing, maturity and harvest period effects to authenticate early-spring Longjing tea using stable isotopes and chemometric analyses. Food Control..

[24] Fang S., Huang W.J., Wei Y., Tao M., Hu X., Li T., Kalkhajeh Y.K., Deng W.W., Ning J. (2019). Geographical origin traceability of Keemun black tea based on its non-volatile composition combined with chemometrics. J. Sci. Food Agric..

[25] Budínová G., Vláčil D., Mestek O., Volka K. (1998). Application of infrared spectroscopy to the assessment of authenticity of tea. Talanta.

[26] Aboulwafa M.M., Youssef F.S., Gad H.A., Sarker S.D., Nahar L., Al-Azizi M.M., Ashour M.L. (2019). Authentication and discrimination of green tea samples using UV–vis, FTIR and HPLC techniques coupled with chemometrics analysis. J. Pharm. Biomed. Anal..

[27] Firmani P., De Luca S., Bucci R., Marini F., Biancolillo A. (2019). Near infrared (NIR) spectroscopy-based classification for the authentication of Darjeeling black tea. Food Control..

[28] Diniz P.H.G.D., Barbosa M.F., De Melo Milanez K.D.T., Pistonesi M.F., De Araújo M.C.U. (2016). Using UV-Vis spectroscopy for simultaneous geographical and varietal classification of tea infusions simulating a home-made tea cup. Food Chem..

[29] Peng T.Q., Yin X.L., Gu H.W., Sun W., Ding B., Hu X.C., Ma L.A., Wei S.D., Liu Z., Ye S.Y. (2021). HPLC-DAD fingerprints combined with chemometric techniques for the authentication of plucking seasons of Laoshan green tea. Food Chem..

[30] Wang L., Wei K., Cheng H., He W., Li X., Gong W. (2014). Geographical tracing of Xihu Longjing tea using high performance liquid chromatography. Food Chem..

[31] Navratilova K., Hrbek V., Kratky F., Hurkova K., Tomaniova M., Pulkrabova J., Hajslova J. (2019). Green tea: Authentication of geographic origin based on UHPLC-HRMS fingerprints. J. Food Compos. Anal..

[32] Pons J., Bedmar À., Núñez N., Saurina J., Núñez O. (2021). Tea and chicory extract characterization, classification and authentication by non-targeted HPLC-UV-FLD fingerprinting and chemometrics. Foods.

[33] Ruíz-Capillas C., Herrero A.M., Jiménez-Colmenero F., Siddiqi K.S., Nollet L.M.L. (2018). Flow Injection Analysis-Tandem Mass Spectrometry. Fingerprinting Techniques in Food Authentication and Traceability.

[34] Sun J., Chen P. (2011). A flow-injection mass spectrometry fingerprinting method for authentication and quality assessment of Scutellaria lateriflora-based dietary supplements. Anal. Bioanal. Chem..

[35] Huang H., Sun J., Mccoy J., Zhong H., Fletcher E.J., Harnly J., Chen P. (2015). Spectrochimica Acta Part B Use of fl ow injection mass spectrometric fi ngerprinting and chemometrics for differentiation of three black cohosh species. Spectrochim. Acta Part B At. Spectrosc..

[36] Campmajó G., Saurina J., Núñez O. (2022). FIA–HRMS fingerprinting subjected to chemometrics as a valuable tool to address food classification and authentication: Application to red wine, paprika, and vegetable oil samples. Food Chem..

[37] Bedmar À. Non-Targeted Fingerprinting Methodologies for the Authentication of Tea. Application to the Detection and Quantitation of Frauds in Adulterated Tea Samples with Chicory. Final Degree Research Project, Universitat de Barcelona. http://hdl.handle.net/2445/184649.

[38] Pluskal T., Castillo S., Villar-Briones A., Orešič M. (2010). MZmine 2: Modular framework for processing, visualizing, and analyzing mass spectrometry-based molecular profile data. BMC Bioinform..

[39] Massart D.L., Vandeginste B.G.M., Buydens L.M.C., de Jong S., Lewi P.J., Smeyers-Verbeke J. (1997). Handbook of Chemometrics and Qualimetrics. J. Chem. Inf. Comput. Sci..

