# Supplementary material for: High-Throughput Flow Injection Analysis–Mass Spectrometry (FIA-MS) Fingerprinting for the Authentication of Tea Application to the Detection of Teas Adulterated with Chicory
Source: Foods. 2022 Jul 20;11(14):2153. doi: 10.3390/foods11142153 (PMC9320581; doi:10.3390/foods11142153)
Supplement: Supplementary file 1 [file foods-11-02153-s001.zip › foods-1769020-supplementary.pdf]

Supplementary Material

High-Throughput Flow Injection Analysis-Mass Spectrometry (FIA-MS) Fingerprinting for the Authentication of Tea. Application to the Detection of Teas Adulterated with Chicory

Mònica Vilà [1], Àlex Bedmar [1], Javier Saurina [1,2], Oscar Núñez [1,2,*] and Sònia Sentellas [1,2,3]

[1] Department of Chemical Engineering and Analytical Chemistry, University of Barcelona. Martí i Franquès 1-11, E08028, Barcelona, Spain; monicavr2000@hotmail.com (M.V.); alexbedmar1999@gmail.com (À.B.); xavi.saurina@ub.edu (J.S.); oscar.nunez@ub.edu (O.N.); sonia.sentellas@ub.edu (S.S.)

[2] Research Institute in Food Nutrition and Food Safety, University of Barcelona, Recinte Torribera, Av. Prat de la Riba 171, Edifici de Recerca (Gaudí), Santa Coloma de Gramenet, E08921 Barcelona, Spain

[3] Serra Húnter Fellow, Generalitat de Catalunya, Rambla de Catalunya 19-21, E-08007 Barcelona, Spain

* Correspondence: oscar.nunez@ub.edu

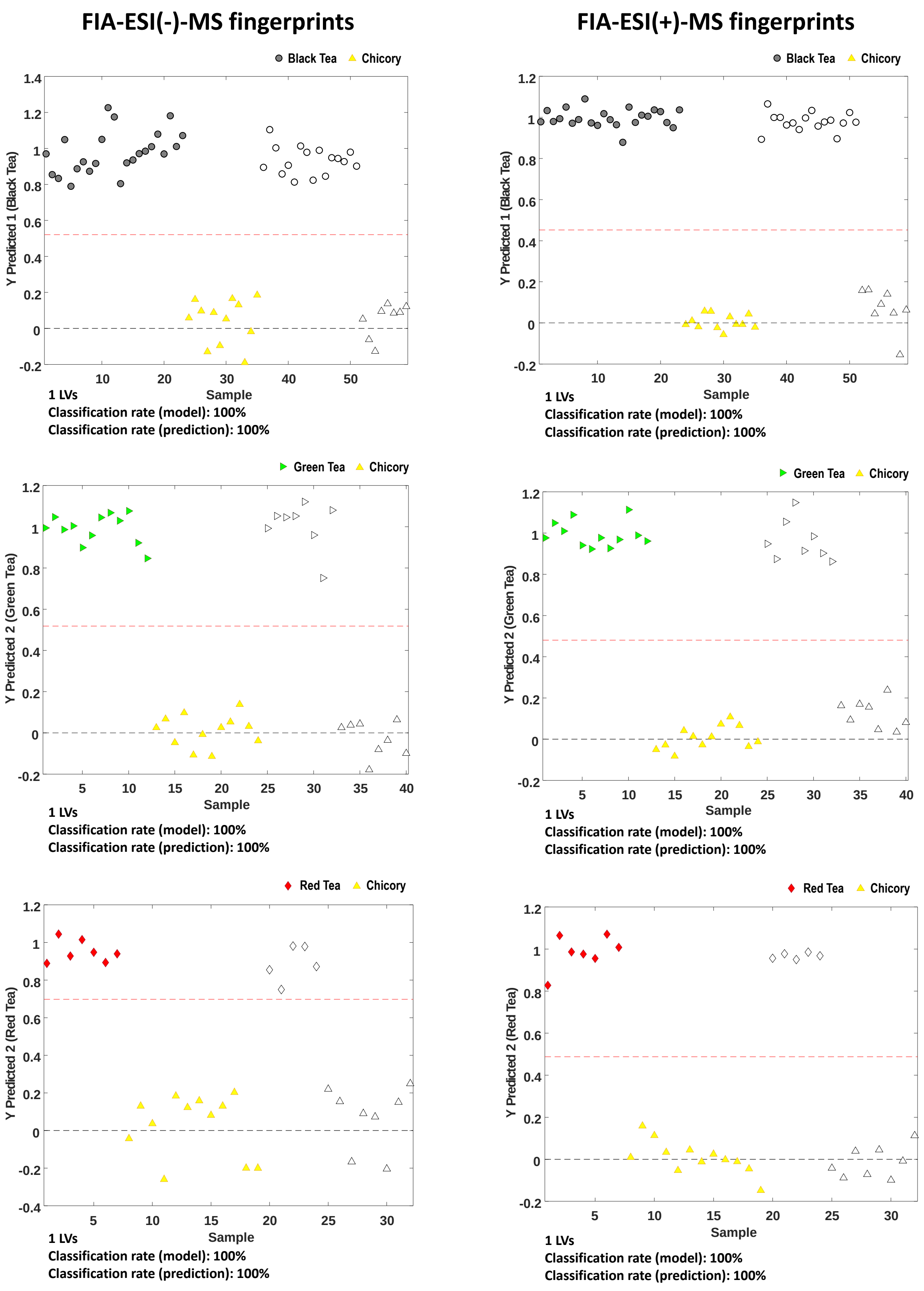
FIA-ESI(-)-MS fingerprints
FIA-ESI(+)-MS fingerprints
Black Tea
Chicory
Y Predicted 1 (Black Tea)
Sample
1 LVs
Classification rate (model): 100%
Classification rate (prediction): 100%
Black Tea
Chicory
Y Predicted 1 (Black Tea)
Sample
1 LVs
Classification rate (model): 100%
Classification rate (prediction): 100%
Green Tea
Chicory
Y Predicted 2 (Green Tea)
Sample
1 LVs
Classification rate (model): 100%
Classification rate (prediction): 100%
Green Tea
Chicory
Y Predicted 2 (Green Tea)
Sample
1 LVs
Classification rate (model): 100%
Classification rate (prediction): 100%
Red Tea
Chicory
Y Predicted 2 (Red Tea)
Sample
1 LVs
Classification rate (model): 100%
Classification rate (prediction): 100%
Red Tea
Chicory
Y Predicted 2 (Red Tea)
Sample
1 LVs
Classification rate (model): 100%
Classification rate (prediction): 100%

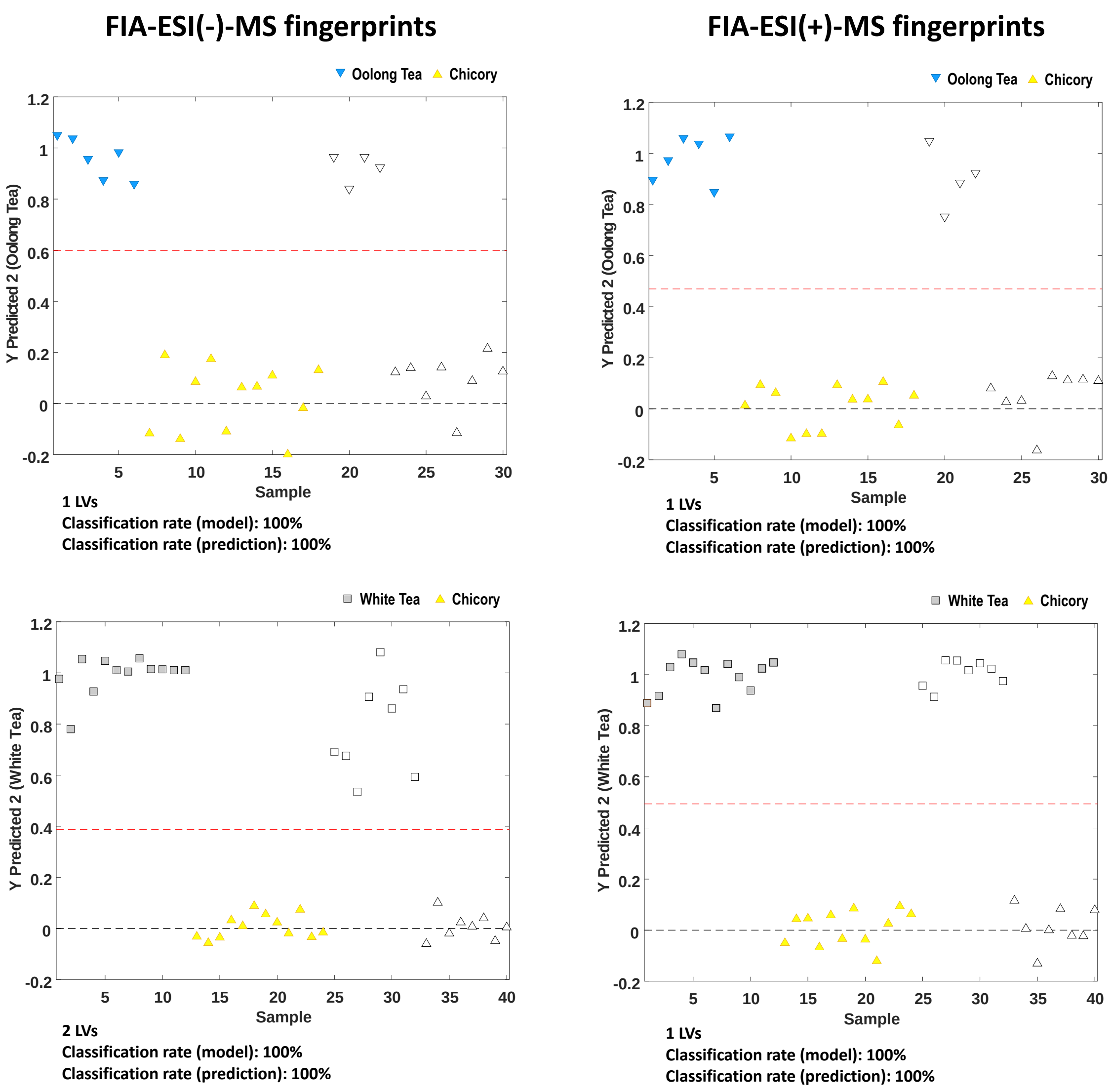

Figure S1. Validation of the paired PLS-DA models of each tea product variety versus chicory when using FIA-MS fingerprints from negative and positive ionization modes as the sample chemical descriptors. Red line establishes the separation threshold between both classes. Filled symbols correspond to the calibration set and empty symbols correspond to the validation set (unknown samples for prediction purposes).

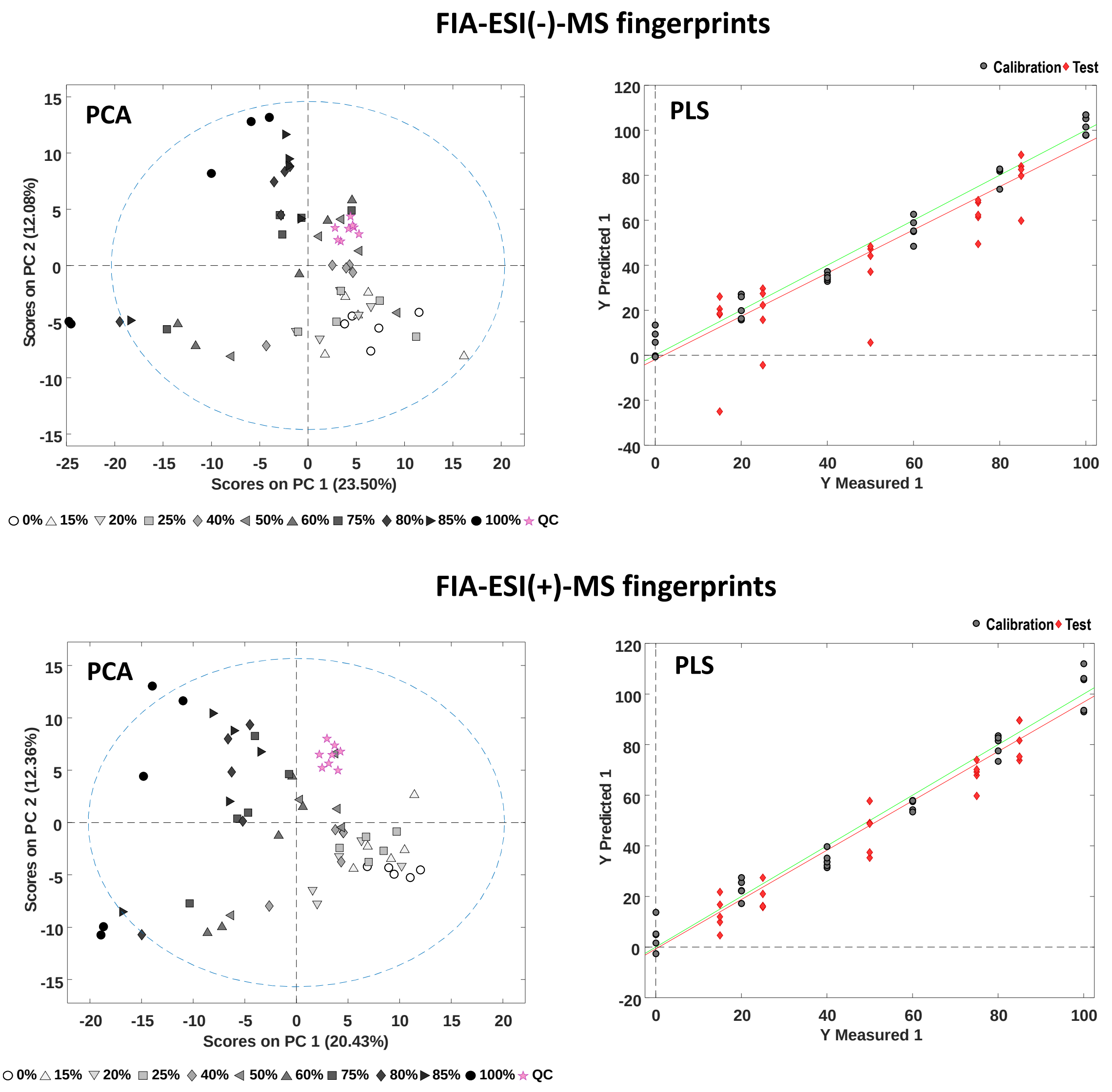

Figure S2. PCA score plots of PC1 versus PC2 depicting the distribution of both calibration and validation sets, and the PLS scatter plots of measured versus predicted percentages of adulterant (chicory) for the black tea adulterated with chicory case, by employing FIA-MS fingerprints in negative and positive ionization mode as sample chemical descriptors.

Table S1. Information about the analyzed samples.

Sample type	Commercial name	Production region	Number of samples (different lots)
Black tea	Ceylon black tea	Sri Lanka	3
	Irish blend	India	3
	Strong English breakfast	Sri Lanka	3
	Yunnan finest tippy, premium	China	3
	India Assam maud	India	3
	Kenya Marnyin	Kenya	3
	Darjeeling ringtong (second harvest)	India	3
	Darjeeling first flush (first harvest)	India	3
	Formosa tarry lapsang souchong	Taiwan	3
	Ceylon quality blend	Sri Lanka	3
	Ceylon Nuwara eliya	Sri Lanka	3
	Korakundah mountain tea	India	3
	Darjelling margaret's hope (first harvest, premium)	India	3
Green tea	Organic gunpowder	China	2
	Pi lo chun (premium)	Taiwan	2
	Sencha (Zhejiang)	China	2
	Lung ching	China	2
	Sencha (premium)	China	2
	Japan Bancha premium	Japan	2
	Japan gyokuro organic	Japan	2
	Mao Feng Jiangsu	China	2
	Assam Jamguri green	India	2
	Lung ching second grade premium	China	2
Oolong tea	Dong ding oolong	Taiwan	3
	Tie kuan yin	China	3
	Milky oolong	China	2
	Special yellow sun	China	2
Red tea	Pu erh Royal (special fermentation)	China	3
	Pu erh Original	China	3
	Pu erh Imperial (manual harvesting)	China	3
	Pu erh Royal Palace	China	3
White tea	Pai Mu tan	China	5
	Silver needles (premium, artisanal production)	China	5
	Imperial Himalayan White Tea	India	5
	Vietnam Mao Feng Organic	Vietnam	5
Chicory	Chicory roots, Valley of Tea	Belgium	5
	Ecologial chicory, Herbes del Molí	Spain	5
	Chicory roots, Health Embassy	England	5
	Chicory roots, Especias Pedroza	Spain	5

Table S2. Blends of black tea samples adulterated with chicory according to the percentage of chicory employed. B1 to B10 refer to ten different black tea product variety samples, and C1 to C4 refer to four different chicory samples. The same procedure was applied with green tea samples.

Adulteration level [%]	Tea	Chicory	Adulteration level [%]	Tea	Chicory
0	B1		60	B1	C2
	B2			B2	C3
	B3			B3	C4
	B4			B4	C1
	B5			B5	C2
15	B6	C1	75	B6	C3
	B7	C2		B7	C4
	B8	C3		B8	C1
	B9	C4		B9	C2
	B10	C1		B10	C3
20	B1	C2	80	B1	C4
	B2	C3		B2	C1
	B3	C4		B3	C2
	B4	C1		B4	C3
	B5	C2		B5	C4
25	B6	C3	85	B6	C1
	B7	C4		B7	C2
	B8	C1		B8	C3
	B9	C2		B9	C4
	B10	C3		B10	C1
40	B1	C4	100		C2
	B2	C1			C3
	B3	C2			C4
	B4	C3			C1
	B5	C4			C2
50	B6	C1			
	B7	C2			
	B8	C3			
	B9	C4			
	B10	C1			
